# A new hollow solenoid receiver compatible with the global double-D transmitter for EV inductive charging

**DOI:** 10.1038/s41598-023-38645-1

**Published:** 2023-07-24

**Authors:** Ahmed A. Shaier, Ahmed A. S. Mohamed, Hamid Metwally, Sameh I. Selem

**Affiliations:** 1 Electrical Power and Machines Department, Faculty of Engineering, Zagazig University, Zagazig, Egypt; 2 Eaton Research Labs, Eaton Corporate, Golden, CO USA

**Keywords:** Electrical and electronic engineering, Renewable energy, Batteries

## Abstract

Inductive power transfer (IPT) technology is a promising solution for charging the electric vehicles (EVs) by enabling an EV to charge its energy storage system (battery) without any connecting wires through magnetic coupling. This paper proposes a new receiver design named Hollow Solenoid receiver that is compatible with the standard double-D transmitter defined by the SAE J2954 standard. A deep design analysis for the proposed Hollow Solenoid receiver is presented to define the optimum design parameters for coil (inductances, number of turns, dimensions, wires, etc.) and ferrite core (dimensions, number, arrangement, etc.). Several WPT3 (11.1kVA) solenoid receiver (SR) designs were presented and analyzed based on efficiency, weight, size, volume, and cost. The performance of the proposed SR was compared with the global Double-D receiver (DDR) presented by the SAE J2954 standard under different lateral and rotational alignment and loading conditions considering coupling factor, transmission power and efficiency, and stray electromagnetic fields (EMFs). The entire IPT system including coils, compensation network, power converters, controls, and battery load was modeled and analyzed for both SR and DDR coils. The results reveal that the proposed SR is compatible with the global DD transmitter at different alignment and loading conditions and is able to transmit the desired power (11 kW) at an efficiency > 85%. Hollow SR design shows the highest efficiency and lowest size, weight, and cost compared to DDR and other designs.

## Introduction

**Figure 1:**
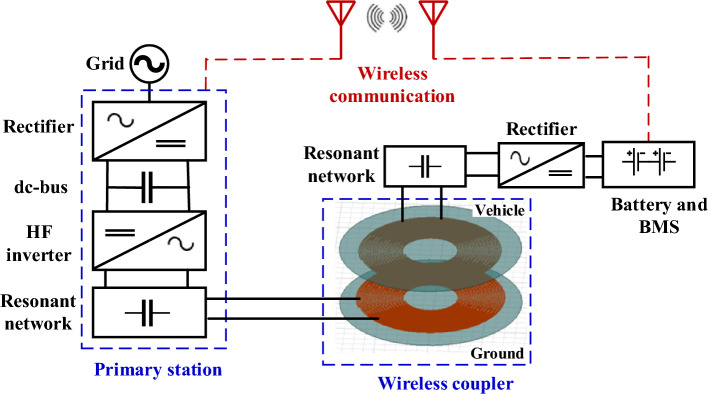
Exemplary loosely coupled IPT system components.

Transportation sector presents a major source of harmful emissions (highest in the U.S.) as it is mainly dependent on fossil fuels which are a non-permanent source of energy and are likely to be depleted over time. Therefore, there is an urgent need to use electric vehicles (EVs) to reduce dependence on fossil fuels and reduce the green-house gases (GHG) emissions. Deploying EV at scale can be hindered if the appropriate charging infrastructure is not available or accessible. Inductive power transfer (IPT) technology shows promising features that enable EV charging during long-term parking as well as movement. IPT is a technique to charge EV batteries over a large airgap distance (100–400 mm) without any physical contact. It has several advantages over plug-in chargers in terms automation, flexibility, safety, maintenance, and convenience. It is also suitable in harsh environmental conditions such as rain, snow, dust, etc.^1^. IPT system consists of two isolated sides; the ground side (transmitter) that contains a primary pad, resonant circuit, high-frequency (HF) inverter, and grid rectifier. The vehicle side (receiver) that contains a secondary pad, resonant circuit, and diode rectifier that feeds the EV battery, as indicated in Fig. 1. The power supply feeds low frequency power to the inverter which converts it to HF power and feeds the transmitter (primary) coil. Electromagnetic fields generated from the primary coil are coupled with the secondary coil to transmit the power at the same supply frequency. The HF secondary power is recertified to charge the EV battery. The primary and secondary sides are talking to each other through a wireless communication link to enable alignment, authentication, control, and paying bills.

The magnetic inductive coupler (primary and secondary pads) is a vital component in IPT system that is responsible for transferring the power from the source to load. Many pad structures and designs have been introduced and discussed in the literature based on to the shape of the generated electromagnetic fields. These structures are divided into three types, Polarized structures, in which the flux components travel horizontally such as double-D (DD) pad and solenoid pad^2^. Structure with vertical flux components is called non-polarized such as rectangular and circular pads^3^. The third structure consists of multiple overlapped coils that are mutually decoupled and can generate both vertical and horizontal flux such as, bipolar and tripolar pads^4^. Rectangular and DD pads for both transmitter and receiver are presented in the SAE J2954 standards for light-duty EV charging. These pads show good advantages in terms of simplicity and performance, which make them suitable for transmitter pad. However, because of the large amount of litz wires and ferrite used in these pads, they show heavy weight, large size, and high cost, which make them inappropriate for a vehicle pad, where space and weight are critical. The solenoid coil is a promising magnetic structure for vehicle pad with very good performance (high coupling factor with high power density and small size) and low cost. It also enables large tolerance of misalignment with medium and large airgaps due to the absence of a zero coupling position because it is a double-sided structure and generates a large non-ionizing radiation^5,6^. The authors in^7^ designed a wireless charging system consists of a flat solenoid coil and an interleaved boost converter. A prototype was built for practical investigation that transmits power of 500 W through an airgap distance of 170 mm. Solenoid coil parameters were optimized and the dc-dc transmission efficiency of 90.1% was obtained. In^8^, the researchers combined a solenoid coil with a rectangular coil to form a transmitter pad. On the other hand, circular and rectangular pads were used separately. A comparison was made between them in terms of variation of coil inductance, the degree of disparity in misalignment, as well as the coupling factor. It was found that when the solenoid is combined and placed in the middle of the rectangular coil, the coupling coefficient is significantly increased. In^5^, an IPT system is designed based on solenoid configuration to transmit the desired power through an air gap of 200 mm. Flux guiding ferrite cores were proposed to increase the coupling coefficient, and a metallic aluminum shielding was used to eliminate leakage electromagnetic fields. The researchers proposed a IPT system based mainly on the solenoid configuration in^9^. This system consists of a flat solenoid on the transmitter side and two solenoids on the receiver side. A system analysis was done to obtain the highest coupling coefficient, and the interoperability principle of the proposed system with DD and bipolar configurations was tested. A 6-kW power transmission is achieved through an air gap of 50 mm, with a horizontal misalignment tolerance of 125 mm.

In^10^, a solenoid coil was designed at both ground and vehicle sides. The factors affecting the coupling coefficient were studied in two ways, mutual inductance and magnetic circuit theories. The ferrite core was also designed to reach the best value of coupling coefficient. In^11^, a solenoid coil structure was used at receiver side while a bipolar structure on the transmitter side. The effect of changing both the number of turns, and the length and width of the ferrite core was analyzed based on the self and mutual inductance, coupling coefficient and magnetic flux density. It was found that by increasing the length or width of the ferrite core, the flux density around the coils increases. Also, increasing the distance between the turns has a bad effect on the field by increasing its value, and significantly reduces the value of self-inductance. In^12^, a new design was proposed at the ground side and derived from the solenoid. It is a combination between central solenoid and rectangular (or square) coil connected in series and is named XPAD. XPAD enables high power transmission through a large airgap with less sensitivity to linear misalignment. It was compared with the conventional solenoid structure and found that it gives a greater coupling coefficient and reduces the stray magnetic field. This improves the performance of the system, but it is more complicated in structure and difficult to manufacture. In^13^, a design for a solenoid to transmit power of 5-kW is presented. The shielding is made of copper in different shapes such as a continuous sheet or multiple small pieces. Optimization was presented for the distances between the ferrite bars to increase the transmitted power. Using the continuous sheet instead of multi-pieces leads to reduce the leakage flux and the eddy current losses by 29%, this makes the system work with high efficiency up to 90%.

The abovementioned studies related to solenoid coil focused on low-power IPT system (< 6 kW) and presents conventional design with solid ferrite core and coil, which doesn’t offer improvements related to size, weight, and cost. Therefore, this study presents a novel design for solenoid coil as a receiver that is compatible with the global DD transmitter. The proposed design offers higher efficiency and lower cost, size, and weight compared to the conventional DD receiver. The main contributions of this manuscript are listed below:

- Proposed a novel design for Hollow Solenoid Receiver coil with a better performance and lower cost/size.
- Developed 3D finite-element models (FEM) for DDT, DDR, and proposed SR considering WPT3 with Z3-classe defined by the SAE J2954 standard.
- Developed circuit models in Simulink including coils, power supply, power converters, resonant network, and battery load for DDT/DDR and DDT/SR systems.
- Estimated the resonant circuit parameters, the operating frequency, and coils passive parameters that enables the system to transmit nominal power with the highest efficiency for each model.
- Designed the proposed SR coil for WPT3 to achieve higher efficiency at lower weight, size, and cost.
- Presented detailed comparative analysis between DDT/DDR and DDT/SR systems considering transmission power, efficiency, and stray EMFs at different alignment (lateral and rotational) and loading conditions.

## Modeling and analysis of WPT3 double-D inductive pads

WPT coils design depends on operating frequency, power levels, coil-to-coil distance (airgap) and the current carrying capacity of conductors forming the coil. In this study, the DD pad recommended by SAE J2954 is used as global pad at ground side to support DD receiver as well as the proposed solenoid receiver. On the vehicle side, two pads are modeled and analyzed: the DD pad provided by SAE J2954 and the proposed solenoid pad. 3D finite-element models (3D FEMs) for all pads DD transmitter (DDT), DD receiver (DDR) and the proposed solenoid receiver (SR) are developed and used to design and optimize the system parameters. Magneto-static solution in ANSYS Maxwell software is used to estimate magnetic parameters and calculate the electromagnetic fields distribution.

### System parameters and design methodology

**Figure 2:**
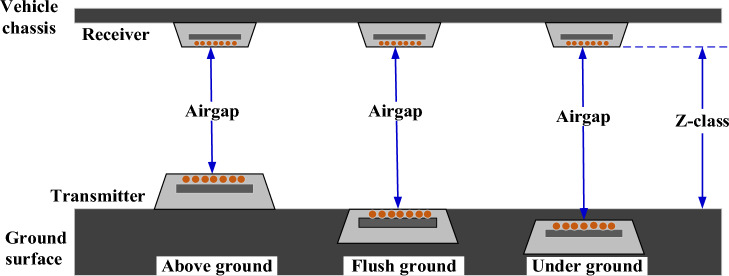
Z-class and airgap for different transmitter pad mounting schemes.

Four classes of power for WPT system are defined by the SAE J2954 standard for light-duty electric vehicles (LDEVs) applications: WPT1 = 3.7 kVA, WPT2 = 7.7 kVA, WPT3 = 11.1 kVA, and WPT4 = 22 kVA. WPT3 power level was targeted in this study to be compatible with the trend from most of the EV manufacturers that considered 11 kW onboard system in most of LDEV models. SAE J2954 provides a reference design for WPT3 including electrical, mechanical, and magnetic, which provides an opportunity for comparison and showing the improvements offered by the proposed design. Such details are not available for WPT4, which still in the early stage. Also, to the authors' knowledge, no study has explored solenoid coupler with WPT3 power level yet. The other classification defined in J2954 depends on ground clearance for each power level to accommodate various models of LDEVs: Z1-class (100–150 mm), Z2-class (140–210 mm), and Z3-class (170–250 mm)^3,14^. The actual perpendicular magnetic separation between the two sides (coil-to-coil distance or airgap) depends on the position of the transmitter pad whether it is under, flush, or above-ground, as indicated in Fig. 2.

**Figure 3:**
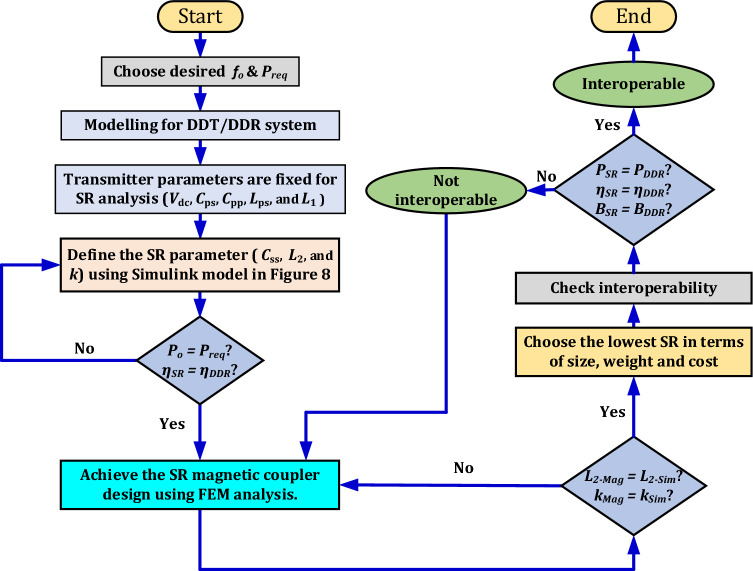
Flowchart of the design methodology.

This study considers on WPT3 power level (11.1 kVA) with Z3-class and the above-ground installation, which means that, the airgap is less than the ground clearance by the thickness of the primary pad. Iterative technique based on trial and error is considered to design the proposed SR coil, which is described by the flowchart in Fig. 3 and summarized below:

- Estimate the passive components parameters of the receiver using a Simulink model: series capacitor (*C*_*ss*_) and magnetic parameters (*L*_*2*_, *k*) that achieves the nominal power with maximum efficiency.
- Develop a magnetic coil in Ansys Maxwell software that achieves the above defined parameters.
- Optimize the design considering different specifications of coils and ferrite cores.
- Evaluate these designs and select the one with the highest efficiency and lowest size, weight, and cost.
- Evaluate performance of the best design of DDT/SR in comparison with DDT/DDR based on: output power (*P*_*o*_), efficiency (*η*), and electromagnetic fields (EMFs) at different alignment and operating conditions.

### Modelling of double-D Pads (DDT and DDR)

**Figure 4:**
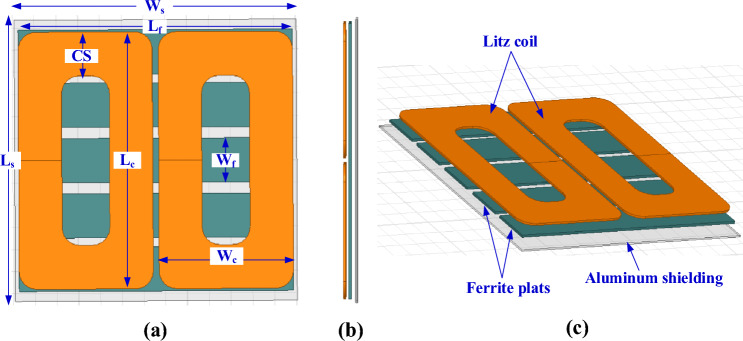
Global DD coil-based transmitter pad for WPT3/Z3 level, (**a**) top view, (**b**) side view, and (**c**) 3D-model.

A 3D finite-element model for the global DDT is constructed considering the specs given in SAE J2954. It contains two similar D-shape single-layer coils made of 5 mm diameter litz wire, as indicated in Fig. 4. This diameter is equivalent to 4 American Wire Gauge (AWG) with construction of 5 × 5 × 3/35/38 according to the datasheet^15^. Turns of the coils are designed as a single turn of copper and the associated number of turns are assigned to the model. This methodology for coil modeling is very popular in 3D FEM in several applications, including inductive charging. It helps to significantly minimize the computational effort and time, and simplify the analysis, while not impacting the results. DDT includes five long plates of ferrite N87 that works as flux concentrators. N87 shows low losses at high frequencies because of its high magnetic permeability and low electrical conductivity that helps to reduce eddy currents. Each plate has a thickness of 6 mm, separated by 20 mm and attached beneath the coil with 1 mm separation distance. An aluminum plate that acts as passive shield is placed under the ferrite layer with a 1.3 mm distance and its thickness is 4 mm^16^. The DDT design dimensions are listed in Table 1.

**Table 1:** Parameters of the DDT global pad.

Parameter	*L*_*c*_	*W*_*c*_	*CS*	*L*_*f*_	*W*_*f*_	*L*_*s*_	*W*_*s*_	*# Turns*
Value	580 mm	308 mm	99 mm	630 mm	100 mm	635 mm	648 mm	6

**Figure 5:**
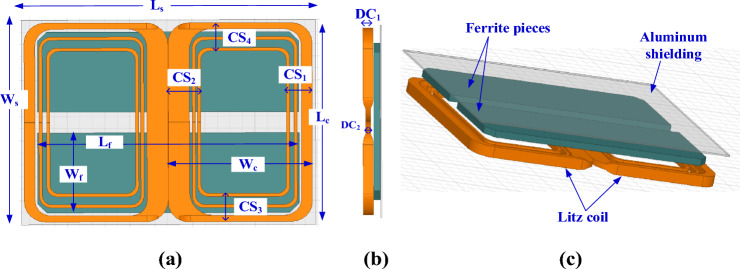
DD coil-based receiver pad for WPT3/Z3.

The DD receiver (DDR) is developed considering the recommendations provided by SAE J2954. It is a union of two identical rectangular coils with non-linear distance between the turns. The external turns are overlapped; however, the internal turns are widely separated to confine the magnetic field lines in the middle of the coil. This coil can be simulated by making each rectangular coil in form of three turns, as indicated in Fig. 5. The coil is supported by two 2 mm long tiles of ferrite separated by 35 mm distance. An aluminum plate is placed on top of the ferrite tiles, at 0.3 mm distance. The DDR parameters and dimensions are listed in Table 2.

**Table 2:** Parameters of DDR/Z3.

Parameter	L_c_	W_c_	CS1	CS2	CS3	CS4	L_f_
Value	330 mm	230 mm	41 mm	53.18 mm	46.5 mm	46.5 mm	418 mm

Parameter	W_f_	L_s_	W_s_	Turns	D_c1_	D_c2_	
Value	132.5 mm	470 mm	390 mm	3 (bifilar)	16 mm	2 mm	

### Magnetic parameter’s estimation and resonant circuit of DDT/DDR-Z3 system

The transmitter and receiver self-inductance (*L*_*1*_ and *L*_*2*_) and coupling factor (*k*) between the transmitter and receiver are obtained at perfect alignment condition, considering the minimum airgap of Z3-class with the above-ground installation. In this case, the airgap is smaller than the ground clearance by 17.7 mm (thickness of transmitter pad). So, the actual airgap for this design is 152.3 mm. The magnetic parameters (*L*_*1*_, *L*_*2*_, *k*) for DDT/DDR system at perfect alignment are extracted from 3D FEM and presented in Table 3. A good correlation can be noticed between the results from the model and the recommended values at the standard SAE J2954^14^.

**Table 3:** Electrical specifications of DDT/DDR WPT3 system for Z3-class.

Parameter	*V*_*dc*_	*C*_*ps*_	*L*_*ps*_	*C*_*pp*_	*L*_*1*_	*k*	*L*_*2*_	*C*_*sp*_	*L*_*s1,*_* L*_*s2*_	*C*_*f*_	*L*_*f*_	*V*_*b*_
DDT/DDR model	800 V	305 nF	22 µH	50 nF	71.72 µH	0.2632	13.52µH	270 nF	250 µH	40 µF	2µH	320 V

Resonant circuits are used in IPT system to compensate for the large leakage reactance related to the large air gap, which leads to improve the transmission power and efficiency of the IPT system. Also, they help to minimize the apparent power supplied by the source by enabling the reactive power demands and achieving unity power factor operation, that provides soft switching for the electronic devices. Many topologies of resonant circuit are reported including: parallel-parallel^17^, series-series^18^, parallel-series^19^, series–parallel^18^, LCL^20^, CCL^21^, and LCC^22^. Comparisons between various configurations are presented in^23–25^.

**Figure 6:**
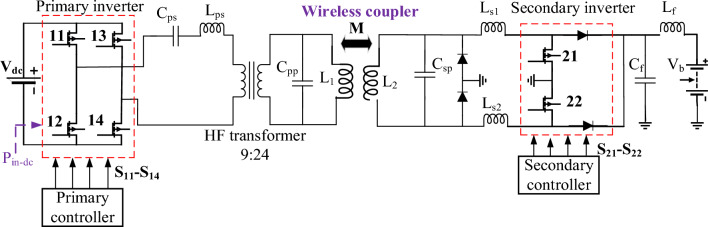
Schematic diagram of DDT/DDR WPT3 system for Z3-class.

**Figure 7:**
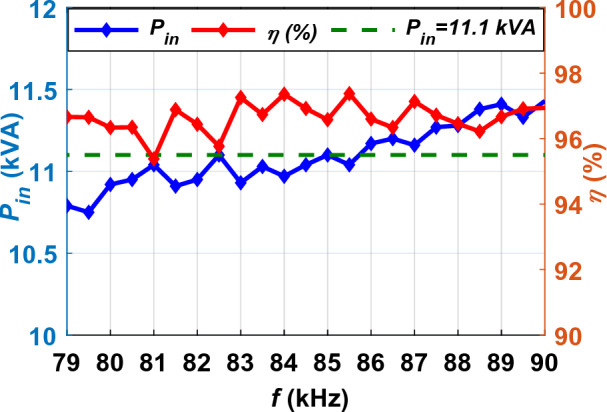
Relationship between *f*, *P*_*in*_ and *η* of DDT/DDR WPT3 system for Z3-class.

SAE J2954 recommends parallel LC resonant circuit for both sides of DDT and DDR, as shown in Fig. 6 with the parameters for Z3-class introduced in Table 3. In this circuit, the frequency of operation is defined as a range from 79 to 90 kHz. To define the proper nominal frequency of operation, the Simulink model for the circuit in Fig. 6 is analyzed within frequency ranging from 79 to 90 kHz by a step of 0.5 kHz. The relationship between input power (*P*_*in*_), dc-dc efficiency (*η*), and operating frequency (*f*) for Z3-class is plotted Fig. 7. As it can noticed, the input power (*P*_*in*_ = 11.1 kVA) is achieved at two frequencies: *f* = 82.5 kHz and *f* = 85 kHz. However, *f* = 85 kHz shows the highest efficiency (*η* = 96.39%) and output power (*P*_*o*_ = 10.70 kW), which is considered in this study.

## Design and analysis of WPT3 solenoid receiver pad

Solenoid receiver (SR) is proposed to be considered at the vehicle side to tresnmit the same power level WPT3 when using the global DDT on the ground side. In this section, several solenoid designs are proposed, evaluated, and compared based on performance and cost.

### Resonant circuit for DDT/SR WPT3

**Figure 8:**
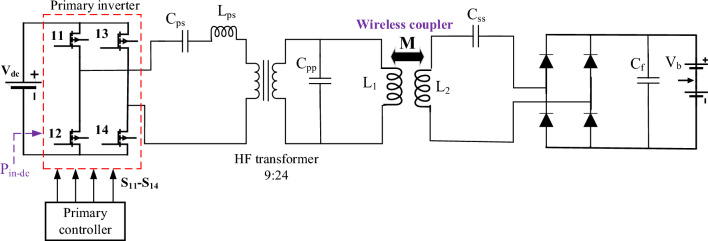
Schematic diagram of DDT/SR of WPT3 system for Z3-class.

In DDT/SR WPT3 system scenario, the same transmitter coil and resonant circuit are considered. For the SR pad, series resonant circuit is used, as depicted in Fig. 8. A Simulink model for the circuit in Fig. 8 is developed and analyzed to define the system operating parameters. The transmitter parameters (*V*_*dc*_, *C*_*ps*_, *L*_*ps*_, *C*_*pp*_) are the same as in DDT/DDR system. The parameters of passive compoennet of SR (*L*_*2*_, *k*, and *C*_*ss*_) are selected such that the system is capable of transferring the nominal power at the perfect alignment conditions, and realize the maximum efficiency (*η*). To verify these conditions, the Simulink model of DDT/SR is analyzed at operating frequency of 85 kHz.

**Figure 9:**
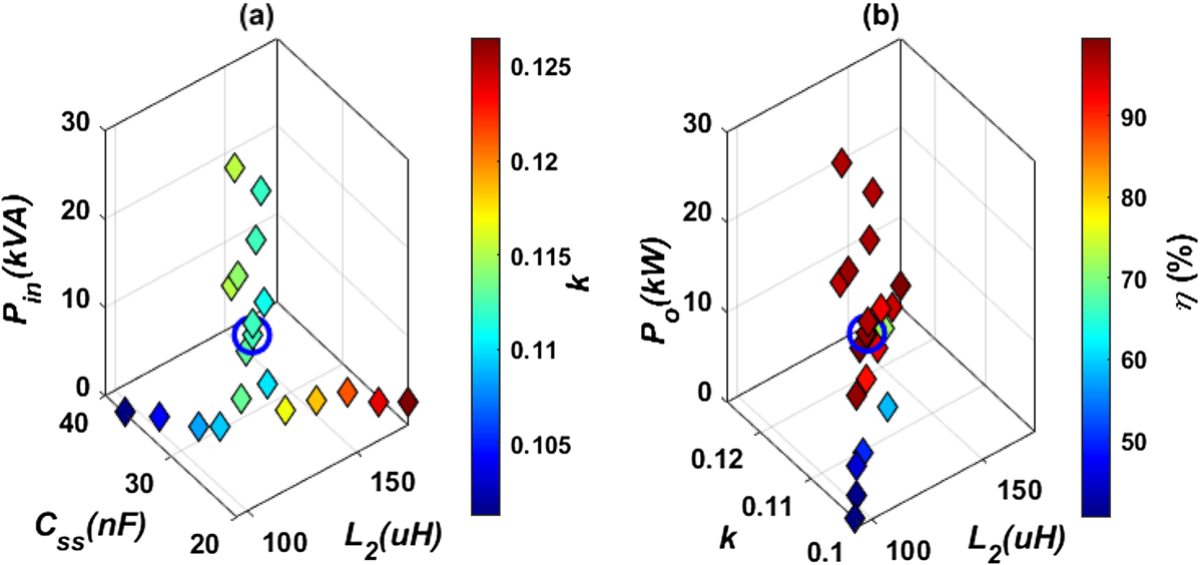
Electrical parameter determination of DDT/SR of WPT3 system for Z3-class @ 85 kHz, (**a**) relationship between* L*_*2*_, *C*_*s****s***_, *P*_*in*_, an d *k*, and (**b**) relationship between* L*_*2*_, *k*, *P*_*o*_,* η*.

At 85 kHz, *L*_*2*_ and *k* are changed and the output power (*P*_*o*_), dc-dc efficiency (*η*), and compensation capacitor (*C*_*s****s***_) are estimated and preseneted in Fig. 9. The values of *L*_*2*_, *k* and the corresponding *C*_*s****s***_ that give 11.1 kVA input power are calculated as indicated in Fig. 9a. These values are used to get a nominal output power (*P*_*o*_ = 10.91 kW) with the highest efficiency (*η* = 98.28%) as shown in Fig. 9b. The selected values are marked with a circle in Fig. 9 and listed in Table 4.

**Table 4:** Electrical specifications of DDT/SR WPT3 system for Z3-class.

Parameter	*L*_*1*_	*k*	*L*_*2*_	*C*_*ss*_	*C*_*f*_	*V*_*b*_
DDT/SR model	70.266 µH	0.11225	125.71 µH	27.826 nF	10 µF	320 V

### Magnetic design of WPT3 solenoid receiver (SR)

**Figure 10:**
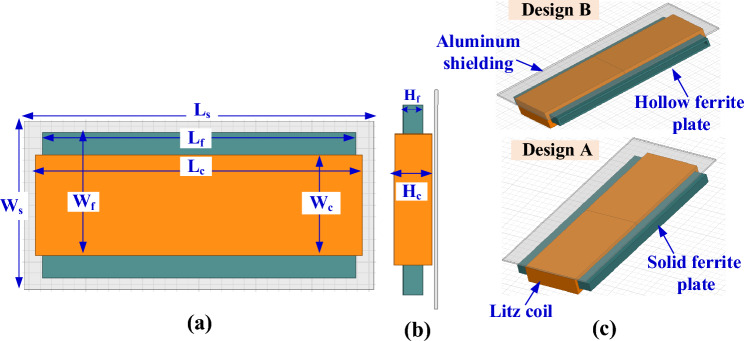
Design A and B of the poposed SR pad for WPT3/Z3, (**a**) top view, (**b**) side view, and (**c**) 3D-model.

The passive parameters of SR coil in Table 4 are used as input the 3D FEM to define and optimize dimensions and specs of coil and ferrite. A 3D FEM for SR was built in Ansys Maxwell Software, which includes a single-layer litz wire with a diameter of 5 mm, as depicted in Fig. 10. The coil in wounded on a solid plate of ferrite with a height *H*_*f*_ to confines and concentrates magnetic flux lines in the area between transmitter and receiver. In addition, a shielding aluminum plate with a thickness of 2 mm is placed above the solenoid litz coil with a 2 mm separation distance.

**Figure 11:**
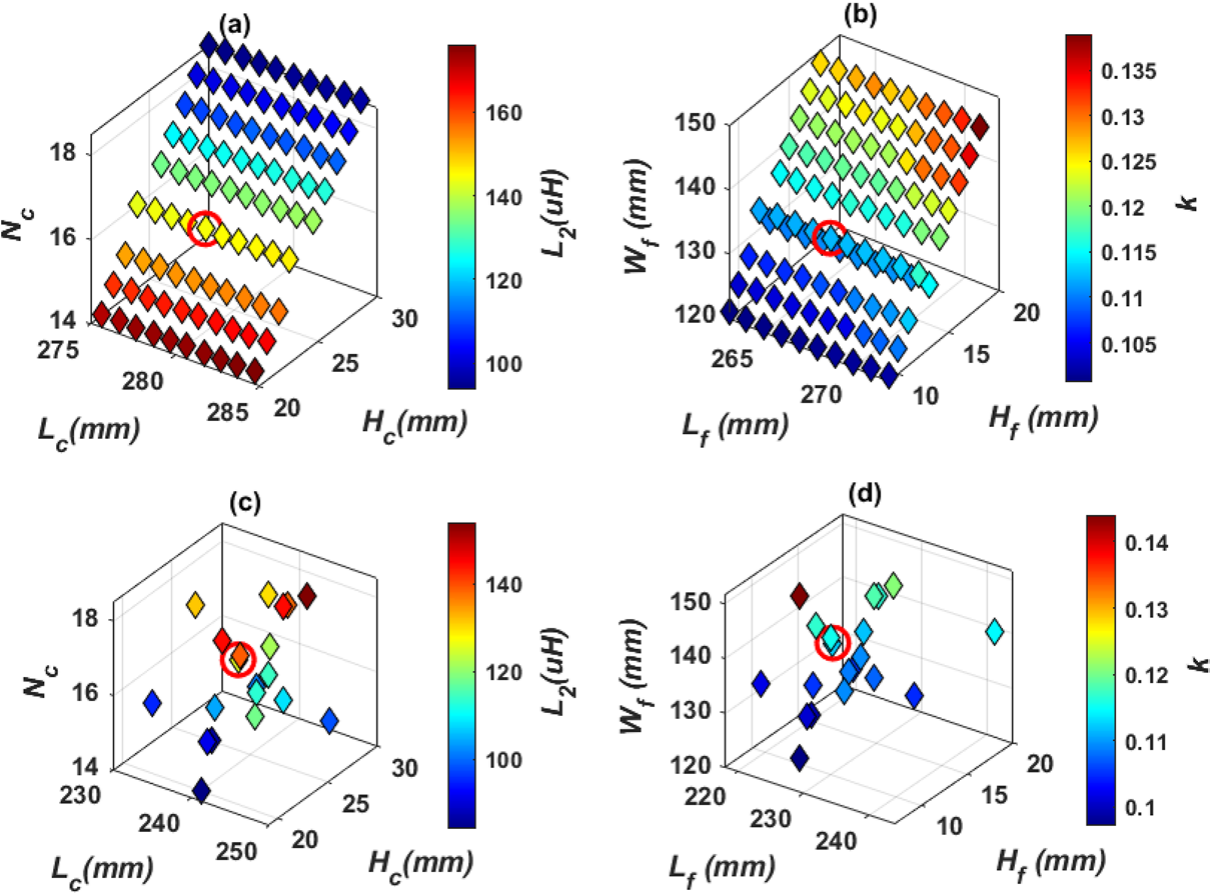
Magnetic design analysis of SR dimensions, (**a**) relationship between *L*_*c*_, *N*_*c*_, *H*_*c*_, and *L*_*2*_, for Design A, (**b**) relationship between *L*_*f*_, *H*_*f*_, *W*_*f*_, and *k*, for Design A (**c**) relationship between *L*_*c*_, *N*_*c*_, *H*_*c*_, and *L*_*2*_, for Design B, and (**d**) relationship between *L*_*f*_, *H*_*f*_, *W*_*f*_, and *k* for Design B.

To obtain *L*_*2*_ and *k* in Table 4, the coil length (*L*_*c*_) is changed from 275 to 284 mm by a step of 1 mm, the number of turns (*N*_*c*_) is changed from 14 to 18 by a step of 1 turn, and the coil height (*H*_*c*_) is changed from 21 to 30 mm by a step of 1 mm. The relationship between these variables is plotted in Fig. 11a. The combination of the values that achieve the desired *L*_*2*_ is marked in red. Another relationship is analyzed to obtain the value of *k*. This value is obtained by changing the length of the ferrite core (*L*_*f*_) from 263 to 272 mm, the width of the ferrite core (*W*_*f*_) from 121 to 148 mm, and the height of the ferrite core (*H*_*f*_) from 9 to 18 mm. A relationship is drawn between these variables and the required *k* is obtained as shown in the circulated point in Fig. 11b.

To minimize the weight, size and cost of SR, design B is proposed which includes a hollow ferrite core instead of using a sloid core., as indicated in Fig. 10c. Same analysis for *L*_*2*_ and *k* are repeated for Design B and presented in Fig. 11c,d. The final specs of SR-Design A and SR-Design B are listed in Table 5.

**Table 5:** Dimensions of the SR designs that show highest power and efficiency for WPT3/Z3 level.

Parameter	*L*_*c*_	*W*_*c*_	*H*_*c*_	*L*_*f*_	*W*_*f*_	*H*_*f*_	*L*_*s*_	*W*_*s*_	*# Turns*
SR-design A	279 mm	89 mm	24 mm	267 mm	133 mm	12 mm	299 mm	149 mm	16
SR-design B	241 mm	95 mm	23 mm	229 mm	142.64 mm	11 mm	261 mm	165 mm	17
SR-design C	319 mm	83 mm	30 mm	138.5 mm	123 mm	18 mm	339 mm	148 mm	15
SR-design D	326 mm	83 mm	25 mm	142 mm	127.11 mm	13 mm	346 mm	143 mm	15
SR-design E	246 mm	95 mm	23 mm	144 mm	60 mm	11 mm	266 mm	160 mm	17
SR-design F	284 mm	89 mm	25 mm	134 mm	75 mm	13 mm	304 mm	149 mm	16
SR-design G	103 mm	65 mm	31 mm	192.5 mm	91 mm	19 mm	202.6 mm	113 mm	24
SR-design H	103 mm	65 mm	31 mm	195 mm	91 mm	19 mm	205 mm	113 mm	24
SR-design I	103 mm	65 mm	31 mm	196.8 mm	31 mm	19 mm	206.8 mm	113 mm	24
SR-design J	103 mm	65 mm	31 mm	200.78 mm	28 mm	19 mm	210.7 mm	113 mm	24
SR-design K	103 mm	65 mm	31 mm	197.7 mm	16 mm	19 mm	207.7 mm	113 mm	24
SR-design L	103 mm	65 mm	31 mm	199.2 mm	16 mm	19 mm	209.2 mm	113 mm	24

**Figure 12:**
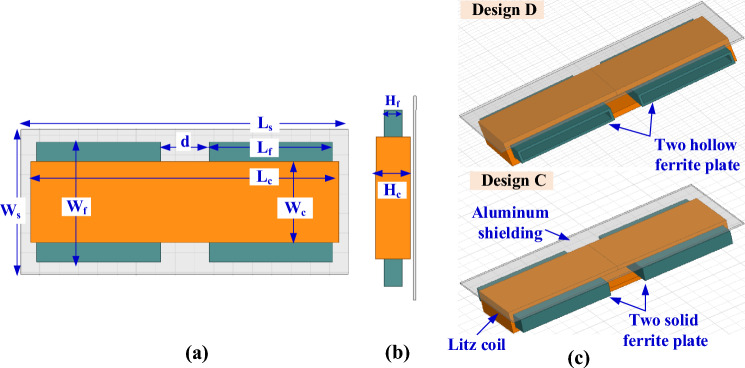
Solenoid receiver pad for Design C and Design D for WPT3/Z3 level, (**a**) top view, (**b**) side view and (**c**) 3D-model.

**Figure 13:**
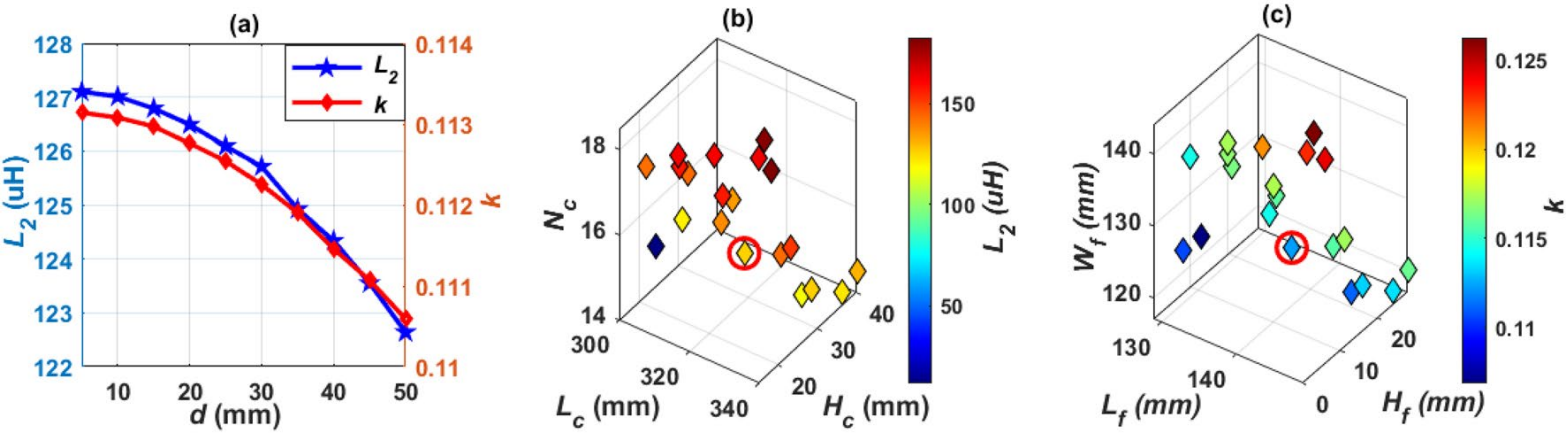
Analysis of SR-Design C dimensions, (**a**) relationship between *d*, *k*, and *L*_*2*_, (**b**) relationship between *L*_*c*_, *H*_*c*_, *N*_*c*_, and *L*_*2*_, and (**c**) relationship between *L*_*f*_, *H*_*f*_, *W*_*f*_, and *k*.

Another Design C can be achieved by using two solid ferrite plates separated by a distance (*d*), as depicted in Fig. 12. The distance *d* is changed from 5 to 50 mm by a step of 5 mm, as shown in Fig. 13a, and the values of *L*_*2*_ and *k* are calculated. It is concluded that increasing *d*, decreases the value of *L*_*2*_ and *k*. The design dimensions that are shown in Fig. 12a,b are changed together to obtain the required value of *L*_*2*_ and *k* that gives the maximum power at the highest efficiency, which was previously mentioned in Table 4. The relationship between these dimensions is illustrated in Fig. 13b,c and the point representing the desired dimensions is marked with a circle. The final dimensions of SR-Design C are listed in Table 5.

**Figure 14:**
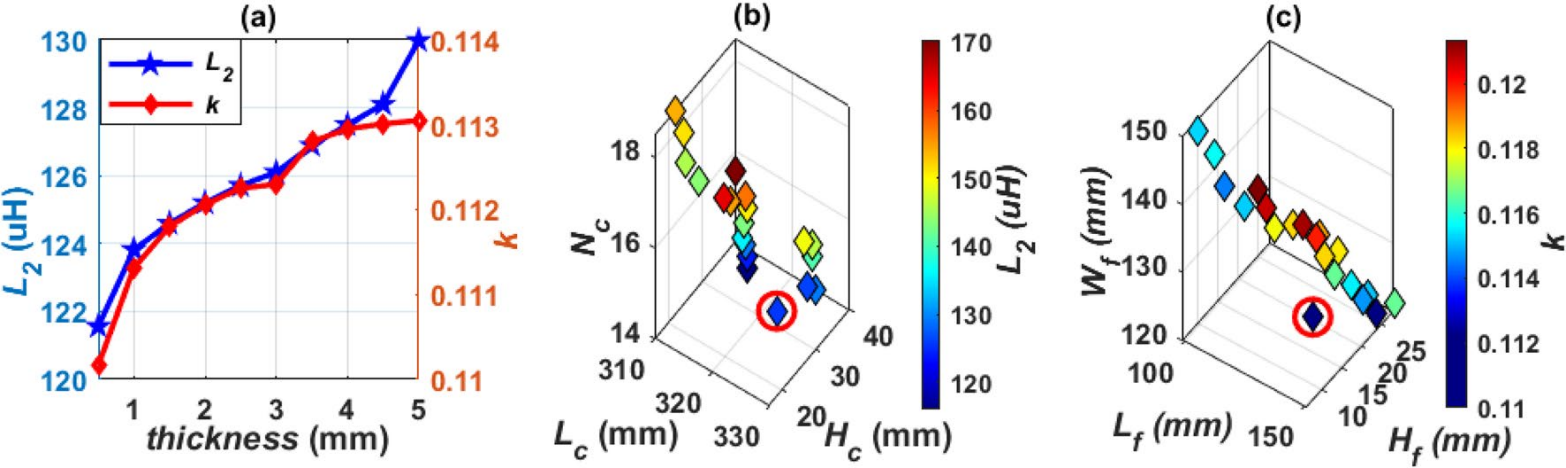
Analysis of SR-Design D dimensions, (**a**) relationship between *thickness*, *k*, and *L*_*2*_, (**b**) relationship between *L*_*c*_, *H*_*c*_,* N*_*c*_, and *L*_*2*_, and (**c**) relationship between *L*_*f*_, *H*_*f*_, *W*_*f*_, and *k*.

Design D is proposed by using two hollow ferrite cores. The 3D-model of this design is depicted in Fig. 12c. The value of *d* that achieve the desired value of *L*_*2*_ and *k* is equal to 30 mm for both Design C and Design D. The relationship between *L*_*2*_ and *k* is studied in the case of using hollow ferrite cores, where the thickness of the cores is changed from 0.5 mm to 5 mm by step of 0.5 mm. It is concluded that increasing the thickness of the core, the value of *L*_*2*_ and *k* increase, as shown in Fig. 14a. The value of core thickness is chosen as 2.5 mm for all designs. In Fig. 14b,c, the relationships between design dimensions that shown in Fig. 12a,b are represented and the point that gives the desired dimensions is circled. The final dimensions of SR-Design D are listed in Table 5.

**Figure 15:**
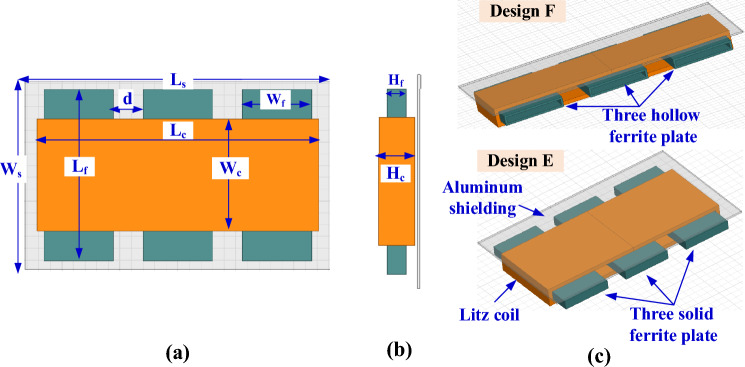
Solenoid receiver pad for Design E and Design F for WPT3/Z3 level, (**a**) top view, (**b**) side view, and (**c**) 3D-model.

**Figure 16:**
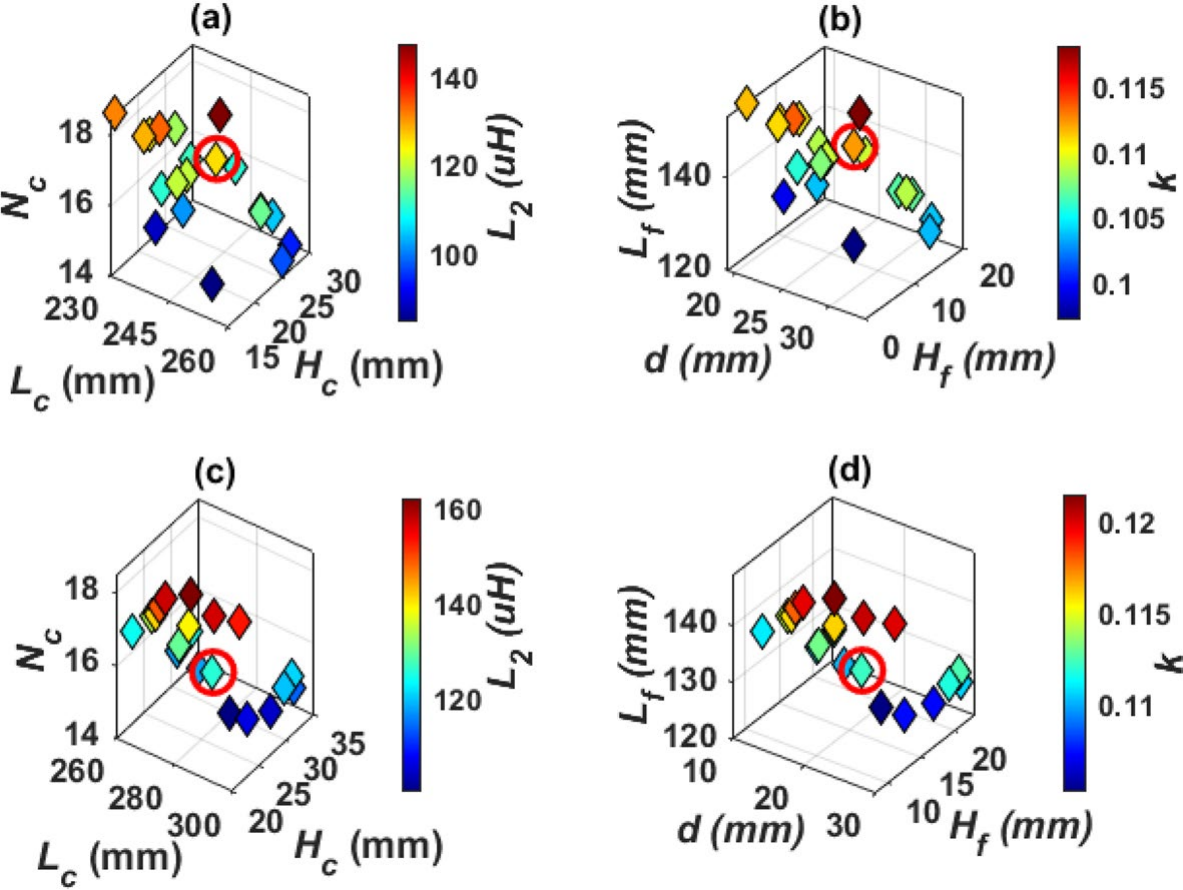
Analysis of SR dimensions, (**a**) relationship between *L*_*c*_, *N*_*c*_, *H*_*c*_, and *L*_*2*_, for Design E, (**b**) relationship between *L*_*f*_, *H*_*f*_, *d*, and *k*, for Design E (**c**) relationship between *L*_*c*_, *N*_*c*_, *H*_*c*_, and *L*_*2*_, for Design F, and (**d**) relationship between *L*_*f*_, *H*_*f*_, *W*_*f*_, and *k* for Design F.

When three solid cores are used inside the solenoid, design E is obtained, as indicated in Fig. 15. The three cores are separated by a distance *d*. The relationship between the dimensions of this system has been studied to obtain the desired values of *L*_*2*_ and *k*. Both (*L*_*c*_, *H*_*c*_,* N*_*c*_, *L*_*f*_, *H*_*f*_, and *W*_*f*_) are changed to achieve the required values (*L*_*2*_ and *k*). The point which represents the required dimensions, is marked with a circle as illustrated in Fig. 16a,b. The dimensions of SR-Design E are listed in Table 5.

If the three cores in design E are hollow, then a solenoid with three hollow cores is obtained, named design F. The 3-D model for this system is shown in Fig. 15c. The change between the system dimensions is studied and the dimensions that achieve the required value of *L*_*2*_ and *k* are obtained. Distance *d* is equal to 27 mm for design E and 23.5 mm for design F. A circle was made on the point that achieves these dimensions, as shown in Fig. 16c,d.

**Figure 17:**
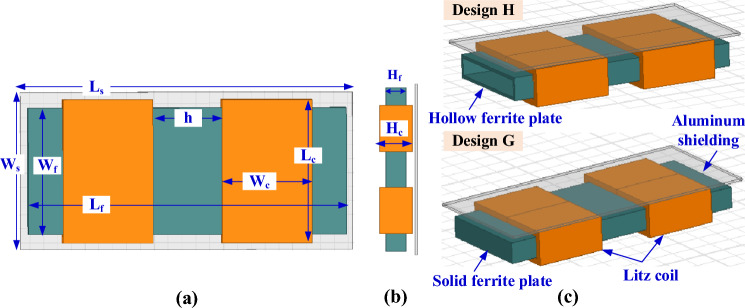
Solenoid receiver pad for Design G and Design H for WPT3/Z3 level, (**a**) top view, (**b**) side view and (**c**) 3D-model.

Another case study is conducted on the SR coil to use multiple coils instead of a single coil. Two identical solenoids are used, connected in series, and separated by a distance (*h*), as shown in Fig. 17. Inside these two solenoids, a solid ferrite core is placed which represents Design G, and a hollow core is placed to represent Design H. The 3D-model for the designs D and H are indicated in Fig. 17c. The distance *h* is changed from 0 to 50 mm by step of 5 mm.

**Figure 18:**
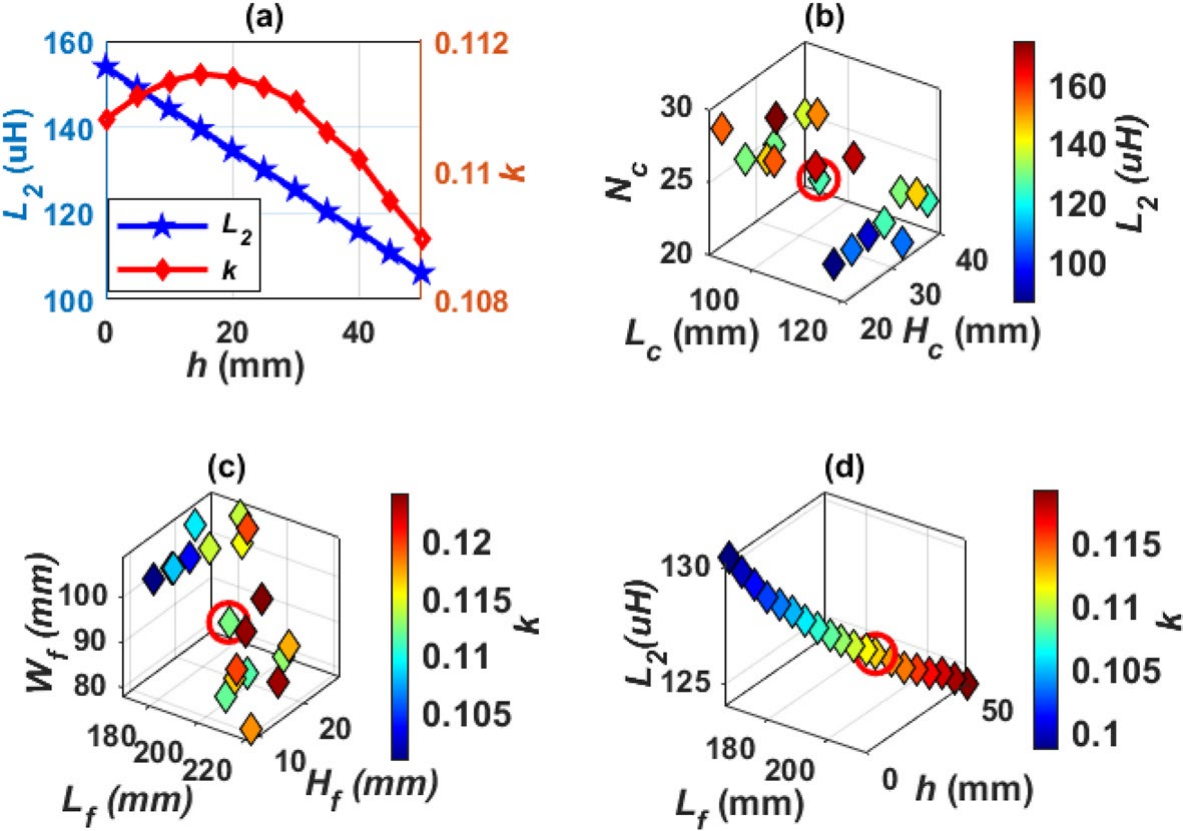
Analysis of SR dimensions, (**a**) relationship between *h*, *L*_*2*_,* k*, for Design G, (**b**) relationship between *L*_*c*_, *H*_*c*_, *N*_*c*_, and *L*_*2*_, for Design G, (**c**) relationship between *L*_*f*_, *H*_*f*_, *W*_*f*_, and *k*, for Design G, and (**d**) relationship between *L*_*f*_, *h*, *L*_*2*_, and *k* for Design H.

The greater the distance between the two coils (*h*) the less self-inductance of receiver coil (*L*_*2*_). The coupling factor increases gradually and reaches its highest value when each of the two SR coils are exactly opposite to the coil side of DDT, then the value of the coupling coefficient decreases as the value of *h* increases as depicted in Fig. 18a. The value of *h* is 32 mm for Design G and 31.8 mm for Design H. For Design G, the coil length (*L*_*c*_) is changed from 90 to 120 mm, the number of turns (*N*_*c*_) from 20 to 29, and the coil height (*H*_*c*_) is changed from 20 to 40 mm. A relationship is drawn between these variables and from which the value of *L*_*2*_ is obtained as shown in circulated point in Fig. 18b. Another relationship is drawn to obtain the value of *k*. This value is obtained by changing the length of the ferrite core (*L*_*f*_) from 186 to 222 mm as well as changing the width of the ferrite core (*W*_*f*_) from 78 to 109 mm and changing the height of the ferrite core (*H*_*f*_) from 9 to 28 mm. A relationship is drawn between these variables and the required *k* is obtained as shown in the circulated point in Fig. 18c. For Design H, the value of the coil dimensions are kept constant as in design G. Therefore, the length of the hollow ferrite core *L*_*f*_ is changed with the distance *h*, and the relationship between these two variables with *L*_*2*_ and *k* is represented as shown in Fig. 18d. The point representing the dimensions required to achieve nominal power transmission with maximum efficiency is marked with a circle. The dimensions of SR-Design G and SR-Design H are listed in Table 5.

**Figure 19:**
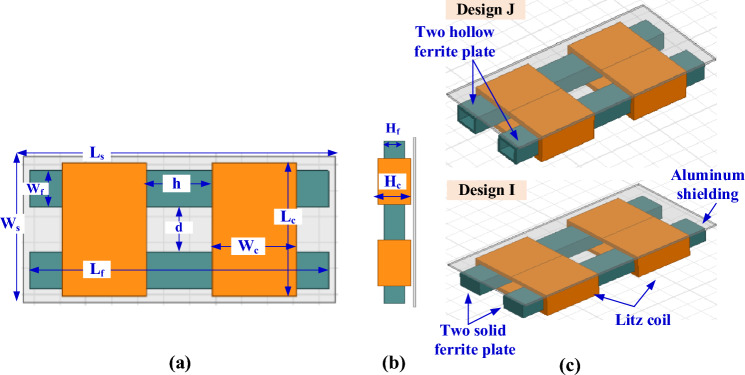
Solenoid receiver pad for Design I and Design J for WPT3/Z3 level, (**a**) top view, (**b**) side view, and (**c**) 3D-model.

**Figure 20:**
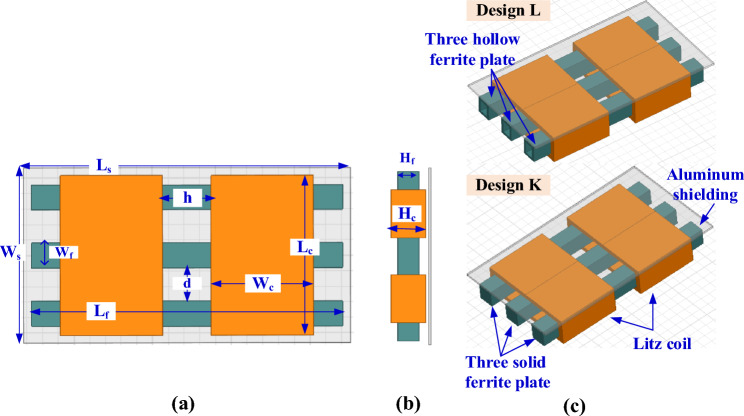
Solenoid receiver pad for Design K and Design L for WPT3/Z3 level, (**a**) top view, (**b**) side view, and (**c**) 3D-model.

When two solenoid coils are used with two solid ferrite cores, this system can be called Design I as depicted in Fig. 19. The coils are separated by a distance *h* and the ferrite cores are separated by a distance *d*. Design J is reached if the two solid ferrite cores are replaced by others hollows as shown in the 3D-model in Fig. 19c. If three solid ferrite cores are used inside the two solenoids, the Design K is obtained as indicated in Fig. 20. Also, the coils are separated by a distance *h* and the ferrite cores by a distance *d*. When the ferrite cores are emptied and used as hollow cores, the Design L is obtained as shown in the 3D model in Fig. 20c. In designs I, J, K and L the coils maintain the same dimensions of Design G in terms of number of turns, length, width and height as indicated in Table 5. The change is only in the dimensions of the ferrite cores and their number in order to obtain the desired value for *L*_*2*_ and *k*. The dimensions of SR-Designs I, J, K and L are listed in Table 5.

**Figure 21:**
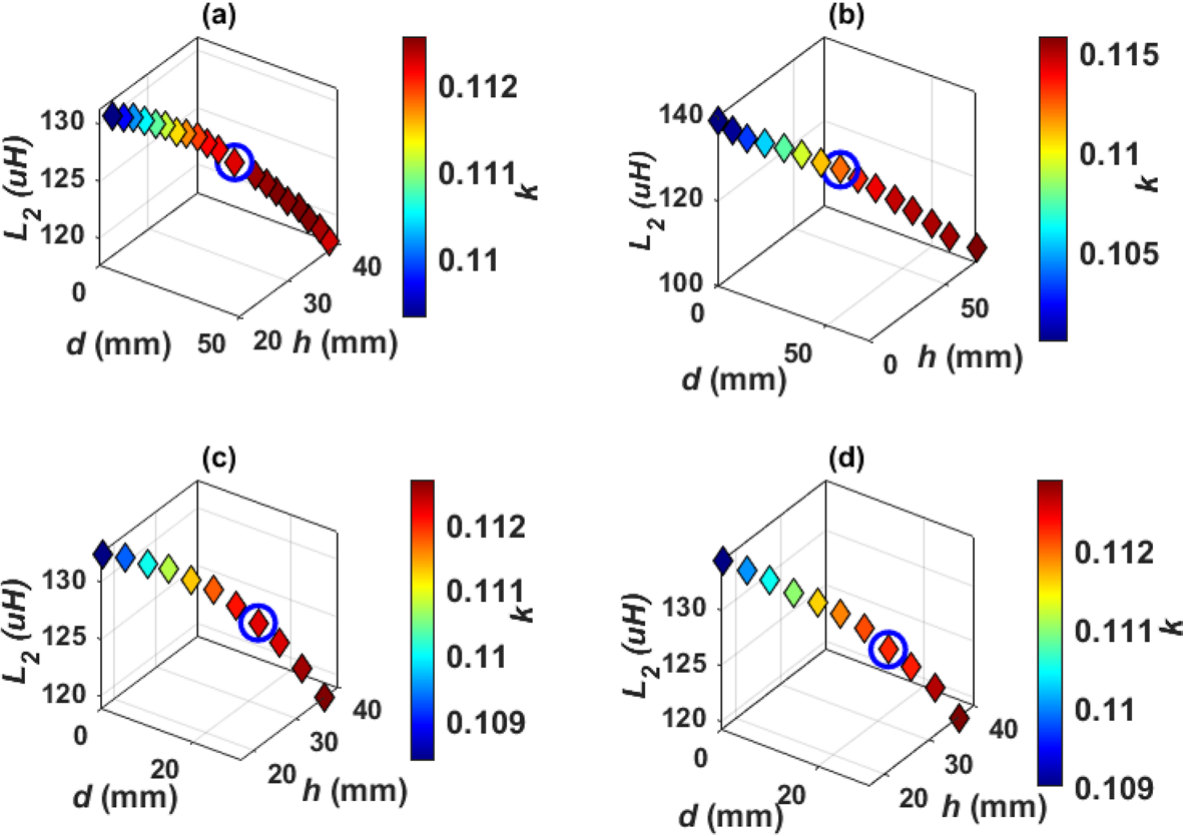
Relationship between *d*, *h*, *L*_*2*_, and *k;* (**a**) for Design I, (**b**) for Design J, (**c**) for Design K, and (**d**) for Design L.

In Design I, the value of *d* is changed from 5 to 47 mm in steps of 2 mm and the value of *h* is changed from 20 to 40 mm in steps of 1 mm. The corresponding values of *L*_*2*_ and *k* are obtained and the relationship between these variables is drawn. The point that gives *d*, and *h* which achieves the desired value of *L*_*2*_ and *k* is obtained and marked with a circle as shown in Fig. 21a, where the values of *d* = 29 mm, and* h* = 31 mm. In Design J, the value of *d* is changed from 5 to 70 mm in steps of 5 mm and the value of *h* is changed from 5 to 70 mm with step 5 mm. The corresponding values of *L*_*2*_ and *k* are obtained and the relationship between these variables is drawn. The point that gives *d*, and *h* that achieves the desired values of *L*_*2*_ and *k* are obtained and marked with a circle as shown in Fig. 21b, where the values of *d* = 35 mm, and *h* = 30 mm.

For Design K, the value of *d* is changed from 0.5 mm to 30.5 mm in steps of 3 mm and h from 16.8 mm to 36.8 mm in steps of 2 mm. For Design L, the distance *d* is changed from 0.5 mm to 30.5 mm in step 3 mm and the *h* from 16.5 mm to 36.5 mm in step 2 mm. The relationship between *d*, *h*, *L*_*2*_, *k* is drawn for both designs and a circle is made on the point that gives the desired values for *L*_*2*_, *k* as in Fig. 21c for design K, and 21(d) for design L. For these four designs, with increase of both distance between the ferrite cores(*d*) and the distance between the two coils (*h)*, the value of *L*_*2*_ decreases and *k* increases.

### Technoeconomic analysis

**Figure 22:**
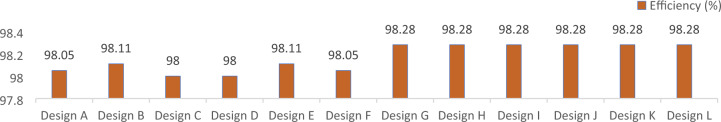
Efficiency values for various solenoid receiver designs.

All proposed designs of SR designs are compared to select the best and most cost-effective one. It is time to determine the most economic design among different designs proposed in section "Magnetic design of WPT3 solenoid receiver (SR)". The resistance of the SR for all designs is very close and ranges from 0.014 Ω to 0.018 Ω. This makes the change in coil efficiency among various designs very small (98%-98.28%) and can be neglected. Thus, all designs transfer the same power (*P*_*in*_ = 11.1 kVA) with almost the same transmission efficiency as indicated in Fig. 22.

A comparison between various SR designs in terms of size, weight and cost is conducted to determine the most economical design. Knowing the dimensions of the designs mentioned in Table 5, the volumes of the litz coils and ferrite cores are calculated. Using litz wire and ferrite material datasheets^15,26^, their weights are calculated. In case of the hollow ferrite cores, a plastic plate is used instead of air for mechanical stability. The plastic material is starboard high-density polyethylene (S-HDPE). This material is considered as one of the lightest types of plastic, with a density of 0.955 g/cm^3^^27^. Therefore, by known the volume of plastic plate, its weight can be calculated^28^.

**Figure 23:**
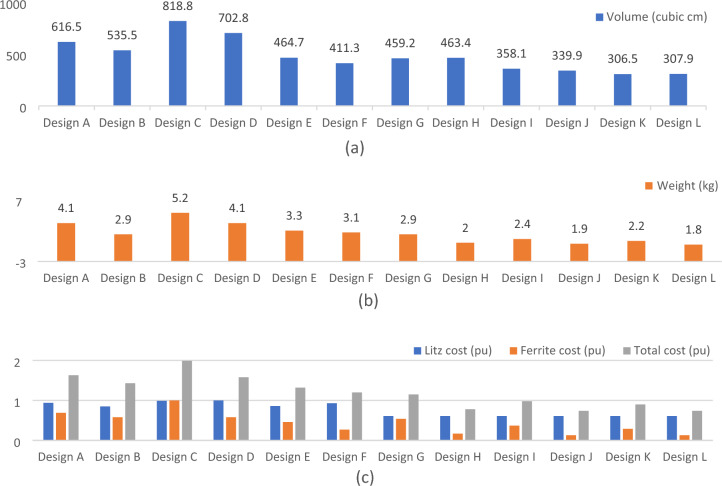
Comparison between volume, weight, and cost for different solenoid receiver designs, (**a**) volume (cm^3^), (**b**) weight (kg), and (**c**) cost (pu).

The chart in Fig. 23a shows the volume of all the proposed designs, where the largest design in terms of size is Design C and the smallest is Design K. The chart in Fig. 23b shows a comparison of weights of the different designs, where the heaviest is Design C and the lightest is Design L. Although the Design K is the least one in size, but it weights higher than the Design L, while the size of the Design L is almost equal to the size of the Design K. Accordingly, Design L can be considered the least in terms of size and weight. The total cost of various SR designs has been calculated and presented in Fig. 23c. Knowing the length of the litz wire and the price per meter mentioned in^29^, the cost of litz wire of each SR design is calculated. Knowing the price per core whose dimensions are (91 × 56 × 10 mm^3^) mentioned in^30^, and the total volume of ferrite cores for each design, the total ferrite cost is calculated. The cost of litz wire and ferrite core represents the total cost for each design. From Fig. 23, it can be noticed that Design L is the lowest in cost. Based on this analysis, it can be concluded that the Design L shows the least size, weight, and cost while showing the highest efficiency (98.28%) compared to other designs.

## Interoperability analysis

Bearing in mind that, the electric vehicles may contain various kinds of receiver pads at the same time that the charging station may contain other types of transmitter pads, these various pads must be work together harmoniously, efficiently and smoothly. The principle of interoperability states that any vehicle must be able to charge its battery from any station, regardless of the pad configuration on both sides. This concept eases the vehicle charging process because the driver does not need to pay attention to the pad type in the vehicle or in the station, therefore the vehicle can be charged from any public station. In this section, the interoperability of the two systems DDT/DDR and DDT/SR-Design L is studied. SR-Design L is chosen because of its features in term of weight, size and cost. The interoperability is measured based on performance indices: coupling factor (*k*), output power (*P*), and dc-dc efficiency (*η*), and stray EMFs^3^. These indices are estimated at perfect alignment as well as lateral and rotational misalignments. According to standard J2954, the system may be subjected to linear misalignments in X-axis direction (*ΔX* =  ± 75 mm) and Y-axis direction (*ΔY* =  ± 100 mm), and angular misalignments such as, rotation around X-axis (*Roll* =  ± 2°), rotation around Y-axis (*Pitch* =  ± 2°), and rotation around Z-axis (*Yaw* =  ± 10°).

### Performance of DDT/DDR and DDT/SR-design L

**Figure 24:**
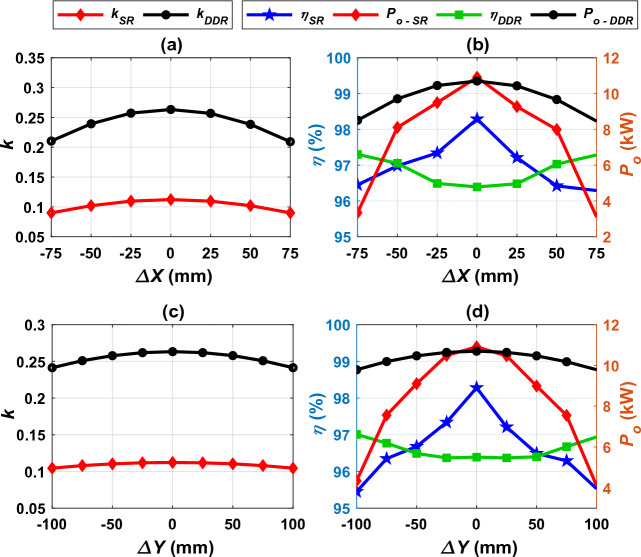
Performance of DDT/DDR and DDT/SR-Design L systems under linear misalignments, (**a**) *k* vs. *∆X*, (**b**) *η, P*_*o*_ vs. *∆X*, (**c**) *k* vs. *∆Y*, and (**d**) *η, P*_*o*_ vs. *∆Y*.

The developed FEMs are analyzed alongside simulation circuit for DDT/DDR and DDT/SR-Design L systems to study the interoperability between them. The performance of both systems considering Z3-class is examined under various linear and angular misalignment conditions. For each system, *k*, *P*_*o*_, and *η* are evaluated and compared at various misalignments. Performance of the two systems under linear misalignments in X- and Y-axes are introduced in Fig. 23 and Table 6. The DDT/SR Design L shows a decrease in coupling coefficient than the DDT/DDR at various misalignment condition in X- and Y-axis directions as shown in Fig. 24a,c respectively. For DDT/SR Design L, the coupling coefficient is significantly lower in misalignment conditions than the ideal alignment position. The power and efficiency of the system is affected by the value of the coupling coefficient, as presented in Fig. 24b,d. DDT/DDR system show robust performance during misalignment in Y-axis direction, which is evident by the small drop in power transfer capability and efficiency from the perfectly aligned condition to the maximum offset, as presented in Table 6. The DDT/SR Design L shows poor power transmission performance at maximum offset in both X- and Y-axis direction. This is shown by the significant drop in the power values, as shown in Table 6. Although the power values are low at maximum offset conditions, the efficiency values remain within the permitted limit for both systems (*η* > 85% for alignment conditions and *η* > 80% for misalignment conditions). When moving in in X-axis direction, both models show large drop in power (71.58% for the DDT/SR-Design L, and 21.02% for the DDT/RDD), because of the large reduction in coupling factor. Nevertheless, efficiency of the two systems remain within the permitted limit, that ranging from 95.45% to 97.28%. Unlike RT/RDD model, the DDT/SR-Design L model is sensitive to linear misalignments.

**Table 6:** Efficiency and power ranges for DDT/DDR and DDT/SR-Design L under various misalignments.

	DDT/DDR (*η_*aligned*_ = 96.39% and *P_*o,aligned*_ = 10.70 kW)				DDT/SR-Design L (*η*_*aligned*_ = 98.28% and *P*_*o,aligned*_ = 10.91 kW)			
	**η_*offset*_ (%)	*Δη%*	**P_*o,offset*_ (kW)	*ΔP*_*o*_*%*	*η*_*offset*_ (%)	*Δη%*	*P*_*o,offset*_ (kW)	*ΔP*_*o*_*%*
*∆X*	97.28	− 0.92	8.45	21.02	96.29	2.02	3.10	71.58
*∆Y*	96.94	− 0.57	9.78	8.59	95.45	2.87	4.10	62.41
*Roll*	95	1.44	7.10	33.64	89.51	8.92	2.41	77.91
*Pitch*	97.19	− 0.82	8.29	22.52	96.02	2.29	4.14	62.05
*Yaw*	97.18	− 0.81	8.81	17.66	96.55	1.96	4.75	56.46

*η_*aligned*_ and *P*_*o,aligned*_ are the efficiency and power at the ideal alignment cases.

**η_*offset*_ and *P*_*o, offset*_ are the efficiency and power at the maximum (worst) misalignment cases.

**Figure 25:**
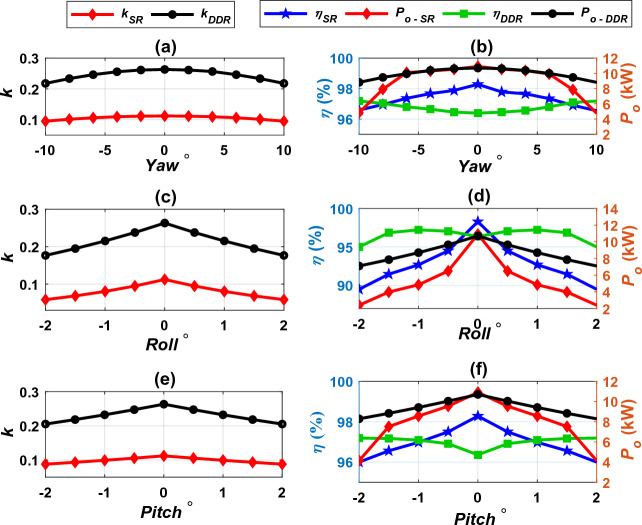
Performance of DDT/DDR and DDT/SR-Design L systems under angular misalignments, (**a**) *k* vs. *Yaw*, (**b**) *η, P*_*o*_ vs. *Yaw*, (**c**) *k* vs. *Roll*, (**d**) *η, P*_*o*_ vs. *Roll,* (**e**) *k* vs. *Pitch*, and (**f**) *η, P*_*o*_ vs. *Pitch*.

Figure 25 presents the performance of DDT/SR-Design L and DDT/DDR under the various angular misalignments (*Yaw *^*o*^, *Roll *^*o*^, and *Pitch *^*o*^). Power and efficiency for both systems are compared in Table 6. The profiles of power and efficiency are nearly constant in the DDT/DDR system for *Yaw *^*o*^ and *Pitch *^*o*^; therefore, this system gives a robust performance. In *Roll *^*o*^, the power is reduced with increasing the value of *Roll *^*o*^. The DDT/DDR system gives a small drop in power (17.66–33.64%) and efficiency (-0.81–1.44%) compared to DDT/SR-Design L which presents a significant reduction in power (56.46–77.91%) and efficiency (1.96–8.92%) at worst misalignment cases. For all angular misalignment cases, efficiency values still within the permitted limits and ranging from 89.51% to 97.19%.

At full alignment case, and when a slight misalignment occurs, the two systems transmit almost the same power with efficiency within acceptable range. In case of DDT/SR-Design L system, the power is reduced at worst offset conditions. Therefore, it can be concluded that, this system is sensitive when misalignments are maximum. So, it can be said that the two systems, DDT/DDR and DDT/SR-Design L are interoperable at ideal alignment and slight misalignment conditions.

**Figure 26:**
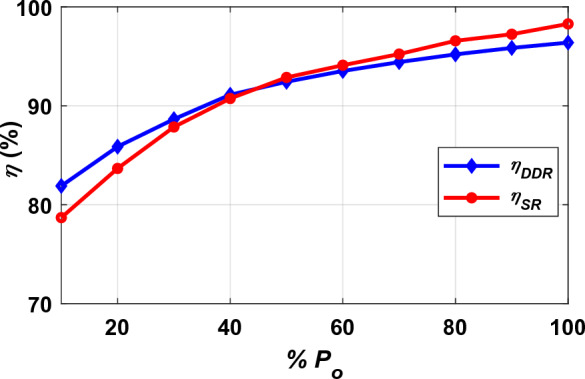
Efficiency (*η*) vs. loading percentage of *P*_*o*_ (% *P*_*o*_).

The effect of variable loading conditions on transmission efficiency (*η*) was studied for both DDT/DDR and DDT/SR-Design L models as depicted in Fig. 26. It was noted that with the increase in the load percentage, the efficiency of each of the two models increases, until the highest efficiency is obtained at full load conditions.

### EMF analysis

The transmitter transmits power over an airgap distance by sending significant quantity of electromagnetic fields (EMFs) in IPT systems. Some of these magnetic fields are coupled with the secondary to express the useful power, whilst the others spread out in the air surrounding the model. If the stray fields overtake the permitted limits, they are likely to generate safety concerns for organisms in close vicinity to the model^31^. These stray magnetic fields have a negative effect on medical devices that can be carried by patients, such as pacemakers as it disrupts their operation^32^. Moreover, they can generate huge induced currents within the vital internal organs of the human, which produces heat stress to body tissues and presents great hazards on human health^33^. Many global organizations have set the permitted safe limits for stray EMFs at various operating frequencies. The International Committee for Non-Ionizing Radiation Protection (2010 ICNIRP) one of these organizations which recommended the permitted safe limits of extrinsic magnetic fields density (*B*) of 27 µT for organisms and 15 µT for pacemakers^14,34^. The guidelines of standard J2954 choose the permitted safe limits mentioned by 2010 ICNIRP for IPT systems. Therefore, as a conservative action, it recommended 15 µT as a public limit around the models to fit with limits of organisms and pacemakers.

**Figure 27:**
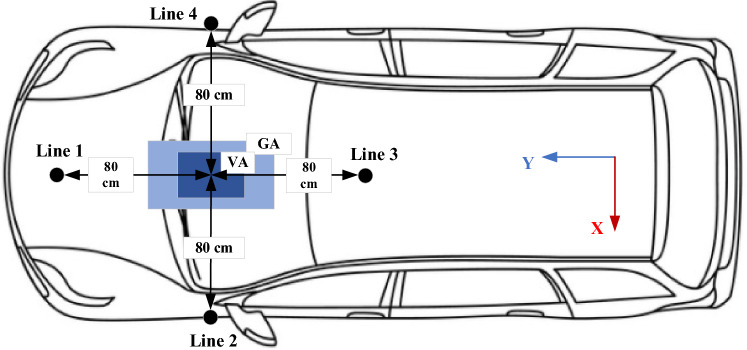
EMFs testing points according to J2954.

The J2954 provides guidelines for measuring the EMFs around the system, which recommended measuring EMFs at 800 mm distance from the meddle of the transmitter pad from the four directions. For doing so, four perpendicular lines are considered in four directions (north, south, east, and west) and started from ground surface to the top of the receiver as depicted in Fig. 27. The two test points in front and behind the vehicle are represented by the lines 1 and 3 respectively, whereas the two sides of the vehicle are represented by lines 2 and 4. Along each line, the magnetic field density value is measured and its maximum value is determined, which is in the mid-distance between the two coils of the model. As a third index of interoperability, the level of electromagnetic fields around the models is taken into account. This level is expected to change with the variation of receiver configurations, misalignment states, and airgap distance. From this point of view, the models must be able to provide the permissible standard safe limits.

**Figure 28:**
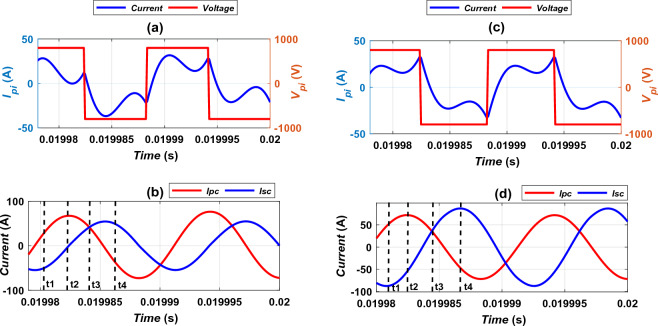
Current’s extraction positions for EMFs models (**a**) *I*_*pi*_ vs. *V*_*pi*_ for DDT/SR-Design L, (**b**) *I*_*Tc*_ vs. *I*_*Rc*_ for DDT/SR-Design L, (**c**) *I*_*pi*_ vs. *V*_*pi*_ for DDT/DDR, and (**d**) *I*_*Tc*_ vs. *I*_*Rc*_ for DDT/DDR.

The electric circuits of DDT/SR-Design L and DDT/RDD models are investigated. The parameters related at ideal alignment condition are considered in this analysis. Output voltage and current for primary inverter (*V*_*pi*_, *I*_*pi*_), transmitter coil current (*I*_*Pc*_), and receiver coil current (*I*_*Sc*_) for DDT/SR-Design L and DDT/RDD systems are compared in Fig. 28. Since magnetic fields are mainly linked to coil currents, four instants of current waveforms are verified. (t_1_, t_2_, t_3_ and t_4_), as indicated in Fig. 28b,d. At each instant, the transmitter current (*I*_*Pc*_) and receiver current (*I*_*Sc*_) are extracted and introduced into the FEMs. The magnetic field distribution is tested for both DDT/SR-Design L and DDT/DDR models, considering the worst case of misalignments. The magnetic field density (B) is measured at all lines from 1 to 4, but due to the similarity in both models, each two opposite lines give identical results. Wherefore, findings at both lines 1 and 2 are taken into account. The *B* values at line 1 (in front of EV) and line 2 (on one side of EV) are shown in Table 7. The instant that shows the largest field distributions is considered for the remaining analysis, which is t_2_ for DDT/SR-Design L and t_1_ for DDT/DDR.

**Table 7:** Worst values of *B* at different instants.

Time	DDT/SR-design L				DDT/DDR			
	*I*_*Pc*_ (A)	*I*_*Sc*_ (A)	*B* at Line 1(µT)	*B* at Line 2(µT)	*I*_*Pc*_ (A)	*I*_*Sc*_ (A)	*B* at Line 1(µT)	*B* at Line 2 (µT)
t_1_	30.58	− 52.11	1.56	3.56	**51**	**− 87.29**	**4.109**	**11.96**
t_2_	**67.19**	**− 4.33**	**2.64**	**4.91**	− 53.89	71.86	3.73	10.42
t_3_	47.45	44.18	1.51	2.36	45.12	38.17	0.60	1.72
**t**_**4**_	− 36.57	49.28	1.81	3.92	− 47.61	86.85	4.00	11.60

Significant values are in bold.

**Figure 29:**
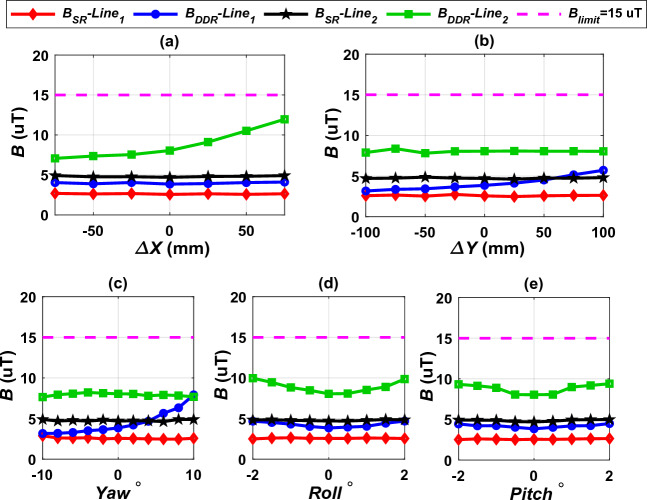
Magnetic flux density distribution in front of the EV (line 1), on the left side of the EV (line 2, (**a**) *B* vs. *∆X*, (**b**) *B* vs. *∆Y*, (**c**) *B* vs. *Yawº*, (**d**) *B* vs. *Rollº*, and (**e**) *B* vs. *Pitchº*.

The measured *B* for DDT/SR-Design L and DDT/RDD models under linear and angular misalignments are shown in Fig. 29. Both models give *B* less than the permitted safe limit with at least 20.26% under various misalignments taking into consideration the uncertainties because of measurements which is generally about 5%^35^. The DDT/SR-Design L system at both lines 1 and 2, produce a magnetic field level of very close value with the DDT/DDR system at line 1. At line 2, the DDT/DDR system produce higher magnetic field level at various misalignments. As shown, the highest values of *B* are much smaller than the accepted safe limit (15 µT). From this it can be said that, the DDT/SR-Design L and DDT/DDR models for WPT3/Z3 are harmonious with the 2010 ICNIRP recommendations for stray magnetic field. Therefore, it can be concluded that, the two models are compatible with each other with a very high degree of safety.

## Conclusion and future work

This study proposes a novel design of Solenoid coil at the receiver pad that is compatible with the standard DD transmitter. An extensive study for designing an appropriate WPT3 solenoid coil for vehicle side with a high efficiency, and small size, weight, and cost was presented. Analytical and 3D FEM are developed and utilized to design and optimize the system/coil parameters. Several solenoid designs were proposed and compared in term of efficiency, size, weight, and cost. SR-Design L with three hollow cores and two coils connected in series shows the highest efficiency and the lowest in size, weight, and cost. Interoperability analysis between the DDT/SR-Design L model and the DDT/DDR model was performed in terms of *k*, *P*_*o*_, *η* and leakage EMFs. The two systems are interoperable in case of perfect alignment and under different misalignment and loading conditions. The two models are harmonious with the safety limits permitted by the international guidelines. *B* values of the two systems are less than 15 µT (acceptable safe limit), taking into account the uncertainty because of the measurements. So, it can be said that the two systems can work together seamlessly and efficiently and this is consistent with the interoperability principle.

The main findings are summarized below:

- Solenoid receiver coil is proposed to support WPT3 and Z3-class vehicles.
- Solenoid receiver coil is interoperable and compatible with the standard DD transmitter coil.
- Hollow solenoid receiver shows significant improvements in size, weight and cost, while maintain high efficiency at different alignment and loading conditions.
- Both DDR and SR-Design L work with a universal DDT and transmit the power required while achieving transmission efficiency within the permissible limits.
- DDT/SR-Design L achieve leakage electromagnetic fields below the permissible limits (15 µT) for both organisms and pacemakers, making them in line with ICNIRP recommendations for electromagnetic fields.

Depending on the analysis, findings, and conclusions presented in this manuscript, numerous research subjects emerge that can be good candidate for future research, that are listed below:

- Conduct extensive experimental testing for the proposed design and the interoperability analysis.
- Explore the compatibility and interoperability of the proposed SR with the global rectangular transmitter recommended in J2954.
- Perform automatic optimization for the proposed solenoid coil parameters to achieve higher efficiency or anti-offset performance.
- Explore the influence of other installation conditions of transmitter pad: flush and under-ground on the interoperability of static inductive systems.
- Study the performance of the proposed solenoid receiver during inductive dynamic charging.

## Data Availability

The data used to support the findings of this study are available from the corresponding author upon request.
